# Quantitative analysis of acetylation in peste des petits ruminants virus-infected Vero cells

**DOI:** 10.1186/s12985-023-02200-1

**Published:** 2023-10-10

**Authors:** Xuelian Meng, Xiangwei Wang, Xueliang Zhu, Rui Zhang, Zhidong Zhang, Yuefeng Sun

**Affiliations:** 1 State Key Laboratory for Animal Disease Control and Preventiony, Key Laboratory of Animal Virology of Ministry of Agriculture, Lanzhou Veterinary Research Institute, Chinese Academy of Agricultural Sciences, Xujiaping 1, Yanchangpu, Chengguan District, Lanzhou, 730046 China; 2 College of Animal and Veterinary Sciences, Southwest Minzu University, Chengdu, Sichuan China

**Keywords:** Acetylation, Post-translational modification, protein-protein interaction, *Peste des petits ruminants* virus

## Abstract

**Background:**

*Peste des petits ruminants* virus (PPRV) is a highly contagious pathogen that strongly influences the productivity of small ruminants worldwide. Acetylation is an important post-translational modification involved in regulation of multiple biological functions. However, the extent and function of acetylation in host cells during PPRV infection remains unknown.

**Methods:**

Dimethylation-labeling-based quantitative proteomic analysis of the acetylome of PPRV-infected Vero cells was performed.

**Results:**

In total, 1068 proteins with 2641 modification sites were detected in response to PPRV infection, of which 304 differentially acetylated proteins (DAcPs) with 410 acetylated sites were identified (fold change < 0.83 or > 1.2 and P < 0.05), including 109 up-regulated and 195 down-regulated proteins. Gene Ontology (GO) classification indicated that DAcPs were mostly located in the cytoplasm (43%) and participated in cellular and metabolic processes related to binding and catalytic activity. Functional enrichment indicated that the DAcPs were involved in the minichromosome maintenance complex, unfolded protein binding, helicase activity. Only protein processing in endoplasmic reticulum pathway was enriched. A protein-protein interaction (PPI) network of the identified proteins further indicated that a various chaperone and ribosome processes were modulated by acetylation.

**Conclusions:**

To the best of our knowledge, this is the first study on acetylome in PPRV-infected host cell. Our findings establish an important baseline for future study on the roles of acetylation in the host response to PPRV replication and provide novel insights for understanding the molecular pathological mechanism of PPRV infection.

**Supplementary Information:**

The online version contains supplementary material available at 10.1186/s12985-023-02200-1.

## Background

*Peste des petits ruminants* virus (PPRV) is a highly contagious virus that profoundly diminishes the productivity of small ruminants worldwide. PPRV, belonging to the genus *Morbillivirus* in the family *Paramyxoviridae* [1], has a negative, non-segmented single-stranded RNA genome that encodes six structural proteins (nucleocapsid protein, N; phosphoprotein, P; matrix protein, M; fusion protein, F; haemagglutinin protein, H; and large polymerase protein, L) in the 3’ to 5’ direction and two non-structural proteins (V and C proteins). PPRV was first reported in the Ivory Coast of West Africa, in 1942 and is currently endemic in Africa, the Middle East and Asia affecting global trade and causing significant economic losses.

The interaction between host and virus is a complex dynamic competitive process. As obligate intracellular parasites, viruses depend on their ability to “hijack” host cellular functions to facilitate their replication and inhibit host antiviral defenses. In contrast, to maintain normal physiological functions, the host utilizes nonspecific and specific immune-based antiviral responses to resist viral invasion, inhibit virus replications, or eliminate virus particles. Therefore, a comprehensive understanding of the molecular mechanism underlying the interaction between the host and PPRV infection may help to further explore the mechanism of PPRV pathogenesis, as well as the development of novel alternative therapeutic approaches.

Protein post-translational modifications (PTMs) affect the functions of proteins by modulating biological processes, protein activity, cellular location and protein-protein interaction (PPI) by transferring modified groups to one or more amino acid residues. To date, more than 450 protein modifications including over 200 PTMs have been identified [2] as dynamic and reversible protein-processing events that play key roles in disease pathogenesis [3–6]. Some PTMs including phosphorylation, acetylation and succinylation potently regulate innate immunity and inflammation in response to viral infection [7, 8].

Of the 20 amino acid residues, lysine is one of the most frequent targets of covalent modifications because it can accept different types of chemical groups [9–14]. Among the lysine PTMs, lysine acetylation is widespread and one of the most well-studied PTMs in both prokaryotes and eukaryotes [2, 15–18]. Lysine acetylation is highly conserved in organisms ranging from bacteria to humans and is particularly important as it affects protein function in multiple cellular processes, including enzyme activity, chromatin structure, localization and PPIs [16, 17, 19–21]. Accumulating evidence indicates that lysine acetylation is an important molecular toggle for protein function [15, 22–24] and is a key regulatory point in mechanisms of both the host antiviral response and virus replication [25–28]. ID1 suppresses FOXO1 transcriptional activity through HDAC4-mediated deacetylation to inhibit foot-and-mouth disease virus replication [28]. RBM10, a splicing factor responsible for SAT1 exon 4 skipping, interacts with dengue viral RNA and RIG-I, and even promotes the ubiquitination of the latter, a crucial step for its activation. It indicated that RBM10 fulfills diverse pro-inflammatory and anti-viral tasks in addition to its well-documented role in the splicing regulation of apoptotic genes [29]. NP acetylation at highly conserved lysine residues affects multiple steps in the replication of influenza A viruses [25]. Lamin acetylation at the nuclear periphery protects against herpesvirus human cytomegalovirus production by inhibiting capsid nuclear egress [26]. However, the extent and function of lysine acetylation in host cells during PPRV infection has not yet been reported.

In this study, we investigated the acetylome of Vero cells infected with PPRV. By combining dimethylation labeling, HPLC fractionation and antibody-affinity enrichment with LC-MS/MS analysis, we systematically and quantitatively compared the acetylome of Vero cells with or without PPRV infection, and calculated the regularity of sequence features around the acetylated sites. We successfully quantified 2641 lysine acetylation sites in 1068 proteins with diverse molecular functions, biological processes and subcellular localizations. Altogether, the results present the first extensive dataset on lysine acetylation in Vero cells infected with PPRV and provide novel insights into the infection mechanism of PPRV.

## Methods

### Cells, virus and infection

**Fig. 1:**
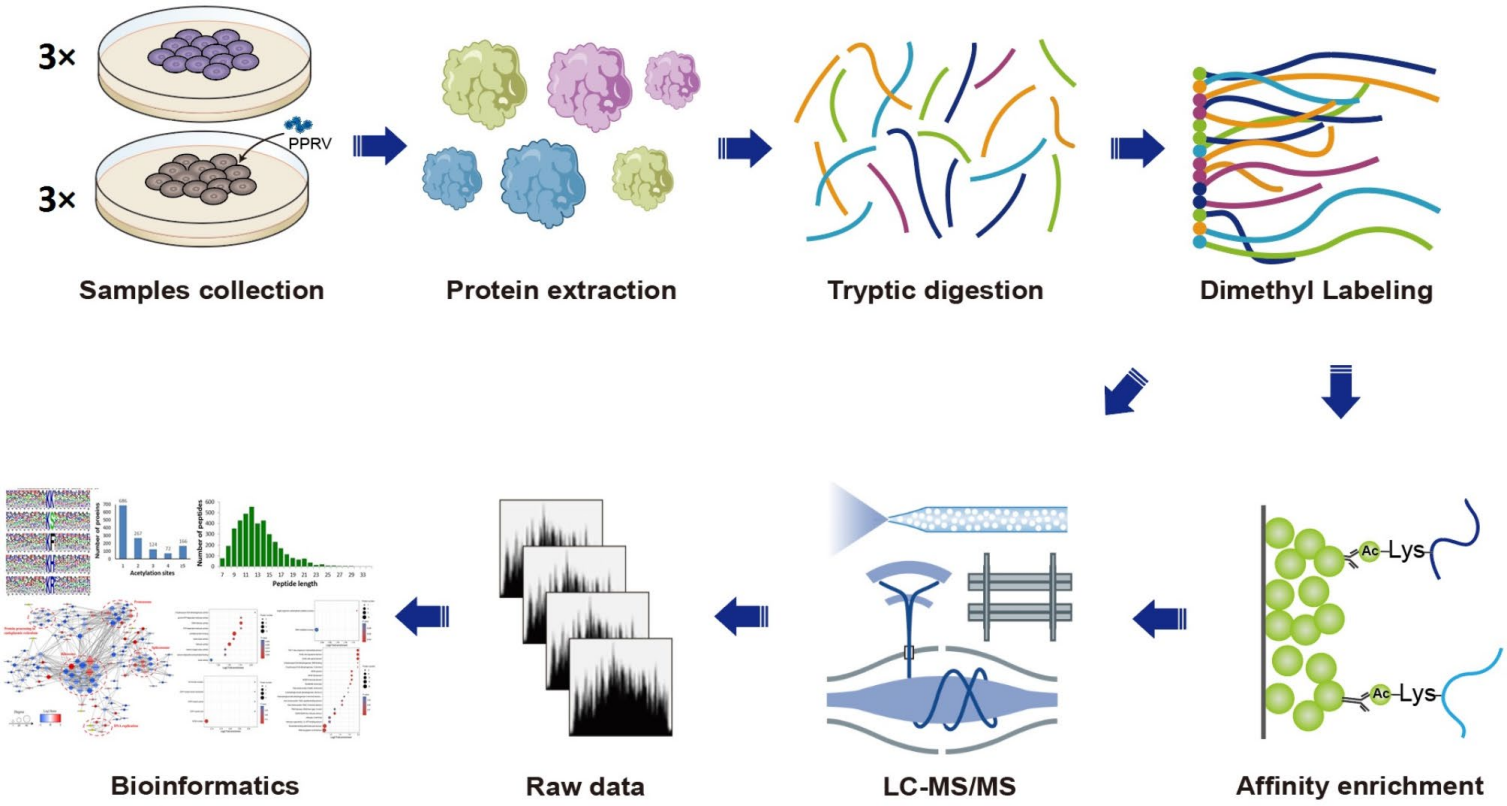
Flowchart of virus infection, proteomic analysis and global mapping of acetylation in PPRV-infected Vero cells

Vero cells were maintained in authors’ laboratory, and cultured in DMEM medium (Sigma Aldrich, St Louis, MO, USA) supplemented with 10% fetal bovine serum, 100 IU/ml penicillin and 100 µg/ml streptomycin and incubated at 37 °C in 5% CO_2_ incubator. The PPRV vaccine strain Nigeria 75/1 preserved in our laboratory was propagated and passaged in Vero cells as previously described [30]. Vero cells seeded in 6-well cell culture plates were infected with PPRV at a MOI of 0.1 or mock-infected with phosphate-buffered saline (PBS, 0.01 M, pH7.4) and incubated at 37 °C for 1 h. The MOI was estimated according to the viral titer of Vero cell line. After adsorption, the virus inoculum was removed and the fresh medium was added to the wells and incubated. In order to determine the sampling time point, the cells were harvested at 24, 48, 72 h post infection (hpi) and analyzed using western blotting and a pan anti-acetyllysine antibody (PTM-104, PTM Biolabs, Chicago, USA). Three independent biological replicates (three parallel experiments) were performed. The flow chart of the present study was shown in Fig. 1.

### Protein extraction

The samples consisted of harvested infected and uninfected cells were washed twice with cold phosphate-buffered saline (PBS) and dispensed in lysis buffer (8 M urea, 1% Protease Inhibitor Cocktail, 3 µM TSA, 50 mM NAM and 2 mM EDTA) then each sample was sonicated on ice. The resulting supernatants were centrifuged at 12,000 rpm for 10 min at 4 °C to remove cellular debris. The protein concentration was determined using a BCA kit according to the manufacturer’s instructions.

### Trypsin digestion and dimethylation labeling

The protein solution was reduced with 5 mM dithiothreitol for 30 min at 56 °C and subsequently alkylated with 11 mM iodoacetamide for 15 min at room temperature in the dark. For tryptic digestion, the protein samples were diluted with urea concentration of less than 2 M by adding 100 mM NH_4_CO_3_. Finally, trypsin was added at 1:50 trypsin-to-protein mass ratio for the first digestion overnight and at 1:100 trypsin-to-protein mass ratio for a second 4 h-digestion at 37 °C. Then, peptide was desalted using Strata X C18 SPE column (Phenomenex) and vacuum-dried. Peptide samples were resuspended in 0.1 M TEAB and labeled in parallel in different tubes by adding CH_2_O or CD_2_O to the control and infected samples, respectively. The reactions were mixed and further treated with NaBH_3_CN (sodium cyanoborohydride), incubated for 2 h at room temperature, then desalted by adding formic acid, and vacuum dried.

### Enrichment for acetylated peptides

Lysine-acetylated peptides were enriched with an agarose-conjugated pan anti-acetyllysine antibody (PTM Biolabs, Hangzhou, China). In brief, dried tryptic peptides re-dissolved in NETN buffer (100 mM NaCl, 1 mM EDTA, 50 mM Tris-HCl, 0.5% NP-40, pH 8.0) were incubated with pre-washed anti-acetyllysine pan antibody-conjugated agarose beads (PTM-104, PTM Biolabs, Hangzhou, China) and incubated at 4 °C overnight with gentle shaking. Then the beads were washed four times with NETN buffer and twice with ice-cold ddH_2_O. The bound peptides were eluted three times from the beads with 0.1% trifluoroacetic acid (TFA; Sigma-Aldrich, St. Louis, USA). The eluted fractions were pooled together and vacuum-dried. The resulting peptides were desalted using C18 ZipTips (Merck Millipore, Billerica, MA, USA) according to the manufacturer’s instructions and dried by vacuum centrifugation, followed by LC-MS/MS analysis.

### LC-MS/MS analysis

The enriched peptides were dissolved in solvent A (0.1% Formic Acid in 2% acetonitrile (ACN)), directly loaded onto a home-made reversed-phase analytical column (1.9 μm particles, 120 A pore, 15 cm length, 75 μm i.d.). The gradient included an increased concentration of solvent B (0.1% Formic Acid in 90% ACN) starting from 9 to 25% for 24 h, followed by 25–40% for 10 min, and reaching to 80% in 3 min, then maintained at 80% for the last 3 min on an EASY-nLC 1000 UPLC (Ultra Performance Liquid Chromatography) system at a constant flow rate of 700 nL/min.

The resulting peptides were ionized and subjected to tandem mass spectrometry (MS/MS) in Q Exactive™ Plus (Thermo Scientific) coupled online to the UPLC using NanoSpray Ionization (NSI) source. The applied electrospray voltage was 2.0 kV. Intact peptides were detected at a resolution of 70,000 with scan range of 350–1800 m/z for full MS scans in the Orbitrap. Peptides were then selected for MS/MS using NCE setting of 28 and ion fragments were detected at a resolution of 17,500. Data-dependent acquisition (DDA), which alternated between one MS scan followed by 20 MS/MS scans, was applied for the top 20 precursor ions with 15 s dynamic exclusion. Automatic gain control (AGC) was used to prevent overfilling of the ion trap and set at 5E4, with a fixed first mass of 100 m/z.

### Database searches

The protein acetylation sites identification and quantification were processed using MaxQuant search engine (v.1.5.2.8) against the *Chlorocebus sabaeus* database (19,228 sequences), concatenated with a reverse database and common contaminants. Trypsin/P was specified as a cleavage enzyme allowing up to 4 missing cleavages. The mass tolerance for precursor ions was set to 20 ppm in the first search and 5 ppm in the main search, and the mass tolerance for fragment ions was set to 0.02 Da. Carbamido-methylation of cysteine (Cys) was specified as a fixed modification, and oxidation of methionine, acetylation on the protein N-terminal, and acetylation on lysine were specified as variable modifications. The false discovery rate (FDR) and the minimum score for the modified peptide were set to < 1% and > 40, respectively. The minimum peptide length was set to 7. All other parameters in MaxQuant were set to default values.

### Bioinformatics annotation analysis

Gene Ontology (GO) annotations were derived from the UniProt-GOA database (http://www.ebi.ac.uk/GOA). The proteins were classified into three categories: biological processes, cellular components and molecular function. Protein domain annotation was performed using InterProScan (http://www.ebi.ac.uk/InterProScan/) based on the protein sequence alignment method, and the InterPro domain database (http://www.ebi.ac.uk/interpro/). The Kyoto Encyclopedia of Genes and Genomes (KEGG) database (http://www.genome.jp/kegg/) was used to annotate and map pathways. GO, protein domain and KEGG pathway enrichment analysis were performed using DAVID Bioinformatics Resources 6.8. Wolfpsort (https://wolfpsort.hgc.jp/), a subcellular localization predication software was used to predict subcellular localization. Amino acid sequence motifs (within ± 10 residues of acetylated sites) were analyzed by Motif-X. Motif-based clustering analyses were also performed, and cluster membership was visualized using a heat map. Functional interaction network analysis was performed using the STRING database (v.11.0), with a high confidence threshold of 0.7 (high confidence), and visualized **using** Cytoscape 3.7.1.

## Results

### Identification of acetylated sites and proteins in PPRV-infected Vero cells

**Fig. 2:**
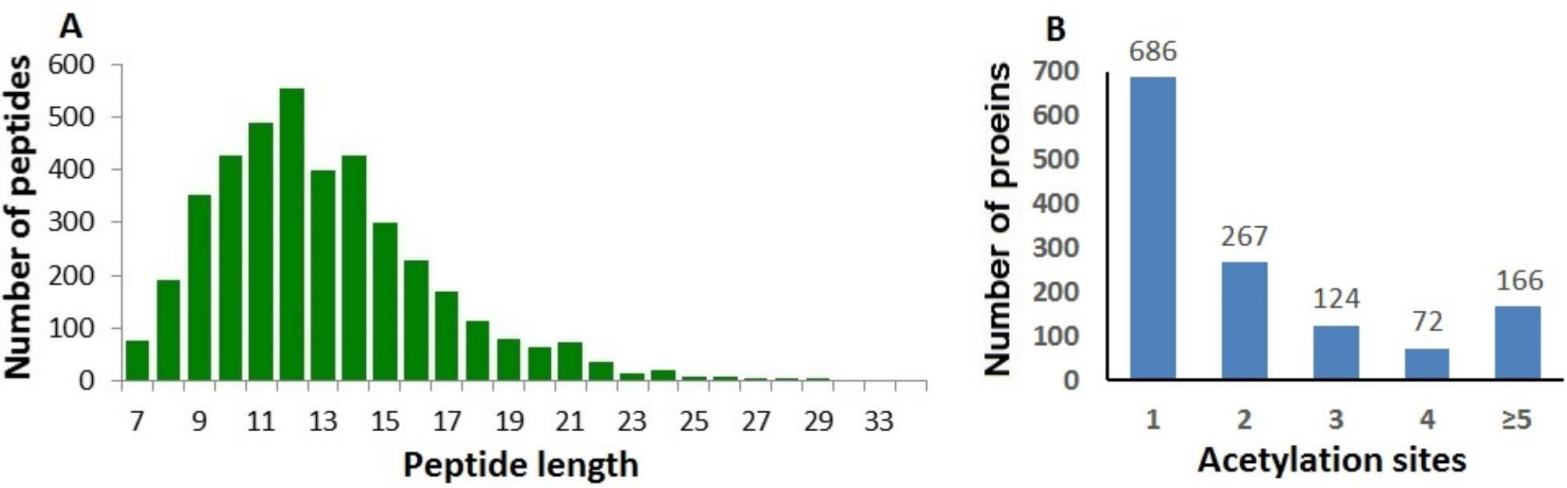
The identification of acetylation proteins and sites in PPRV-infected Vero cells; **A** Peptide length distributions of acetylation profiles; **B** Summary of the acetylated proteins and sites identified

Lysine acetylation can alter the structure and function of proteins involved in diverse biological processes. To explore the acetylated host proteins or pathways involved in PPRV replication, we selected 24 hpi as the time point for quantitative proteomic analysis according to the results of western blotting (Additional file 1: Fig. S1). A combination of iTRAQ based quantitative proteomic and LC–MS/MS was used to identify acetylated proteins and acetylation sites (Fig. 1). The near-zero distribution of mass error and that the errors were predominantly < 0.02 Da (Additional file 2: Fig. S2). Most of the enriched lysine-acetylated peptide lengths were in the size range from 7 to 21 amino acids (Fig. 2A), which was consistent with trypsin cutting at lysine residue sites. The mass spectrometry data have been deposited at the ProteomeXchange (http://proteomecentral.proteomexchange.org/cgi/GetDataset) with dataset identifier PXD025081.

In total, 3229 acetylated sites belonging to 1315 proteins were identified, of which 2641 modification sites in 1068 proteins were quantifiable (Additional file 3: Table S1). Among these quantifiable proteins, approximately 686 (52.16%) contained a single acetylation site, 267 (20.30%) included two acetylation sites, 124 (9.43%) included three acetylation sites, 72 (5.48%) included four acetylation sites, and 166 (12.62%) included five or more than five acetylation sites (Fig. 2B). A total of 107 acetylation sites were found on histone proteins, including 13 sites in H1, 12 sites in H2A, 13 sites in H2B, 9 sites in H3 and 8 sites in H4. These results provide a comprehensive overview of the acetylation events in PPRV-infected Vero cells.

**Fig. 3:**
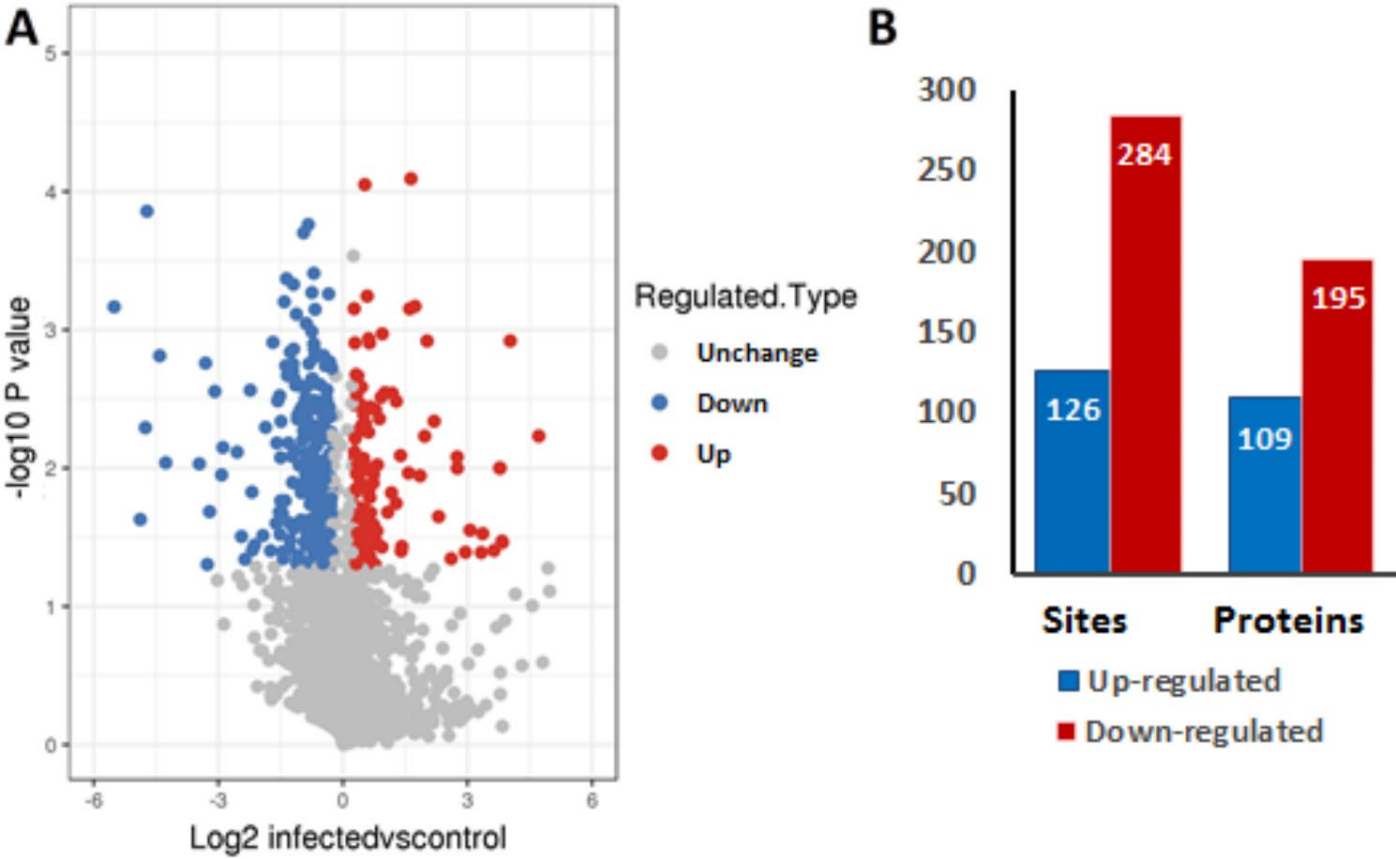
Effects of PPRV on protein acetylation of Vero cells; **A** A volcanic map was constructed using fold-change values and P-values to show acetylated proteins abundance in the two groups, the difference multiple (logarithmic transformation with base 2) was plotted on the abscissa and P-values (logarithmic transformation with base 10) were plotted along the longitudinal axis. Gray dots represented proteins that are not significantly differentially acetylated (P > 0.05). Up-regulated DAcPs were presented as red dots and down-regulated DAcPs were presented as blue dots; **B** Summary of the differentially acetylated proteins and sites (fold change < 0.83 or > 1.2 and P < 0.05)

Based on a threshold of 1.2-fold changes and t test P < 0.05 as standards, 410 acetylated sites in 304 differentially acetylated proteins (DAcPs) were identified (Additional file 4: Table S2), of which 126 acetylated sites in 109 DAcPs were up-regulated, and 284 acetylated sites in 195 DAcPs were down-regulated (Fig. 3). Most DAcPs were modified at a single acetylation site. 79 were modified at multiple lysine acetylation sites, including PDIA4 (7 sites), vimentin (6 sites), plectin (5 sites), nucleolin (NCL, 4 sites) and molecular chaperones. Heat shock proteins (Hsps), including HspA8, HspA5, HspA9, Hsp90AB1 and Hsp90B1, were acetylated at 8, 3, 2, 3 and 3 sites, respectively. Two different sites were acetylated in six paralogous subunits of chaperonin TRiC (also called CCT).

### Functional, subcellular localization and COG classification of differentially acetylated proteins

**Fig. 4:**
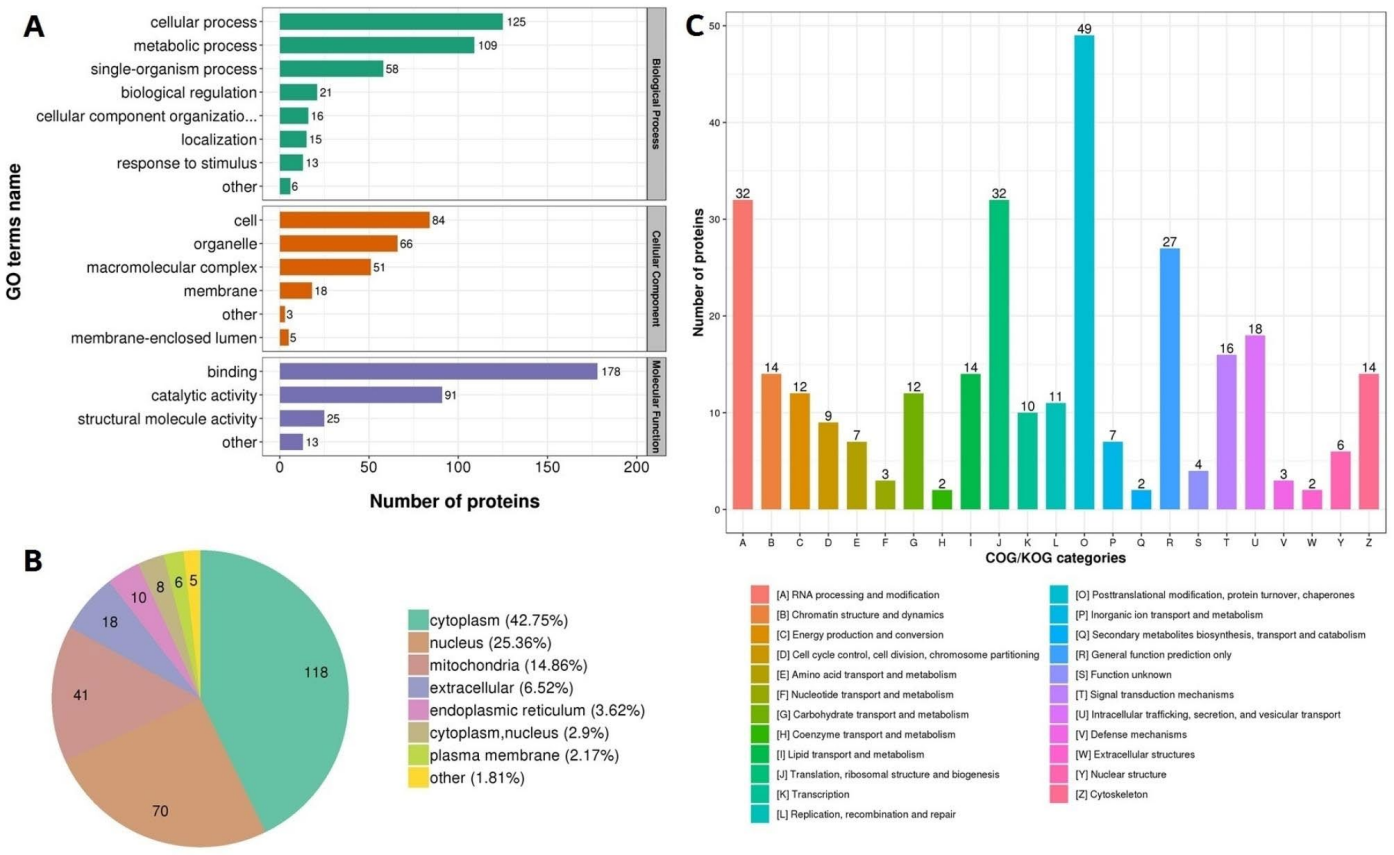
Functional classification of the identified DAcPs in PPRV-infected Vero cells; **A** GO classification of the identified acetylated proteins in three categories: biological process, cellular component and molecular function; **B** Subcellular localization; **C** COG/KOG classification

To better understand the potential functions of acetylation in PPRV-infected cells, all DAcPs were classified by GO analysis based on their biological process, molecular function, subcellular localization, and COG/KOG categories (Fig. 4 & Additional file 5: Table S3).

With regard to biological processes, the acetylated-proteins were primarily associated with either cellular process (34%) or metabolism process (30%) (Fig. 4A). The classification results for molecular function indicated that DAcPs were mostly involved in binding (58%) and catalytic activity (30%). Most of DAcPs were distributed in the cytoplasm (43%), nucleus (25%), mitochondria (15%) or extracellular (7%) (Fig. 4B). Furthermore, based on the results of COG/KOG classification, 271 DAcPs were successfully annotated into 4 categories (Fig. 4C): 39% were involved in cellular processes and signaling; 40% played roles in posttranslational modification, protein turnover, and chaperones; 34% were associated with information storage and processing; and 67% were involved in translation, ribosomal structure and biogenesis, RNA processing and modification. All classification results of the up-regulated acetylated proteins were similar to those of the down-regulated acetylated proteins.

### Motifs analysis of acetylated sites

Amino acid residues surrounding the central lysine acetylation residues have specific patterns and preferences in both eukaryotes and prokaryotes [31]. Thus, to determine the characteristics of acetylated lysine in PPRV-infected Vero cells, the conserved motif surrounding the specific acetylated sites was investigated using the Motif-X program with a significance threshold of P < 0.000001. The amino acids flanking the acetylated sites were matched to the whole size, and the motif enrichment was illustrated in the form of a heat map.

**Fig. 5:**
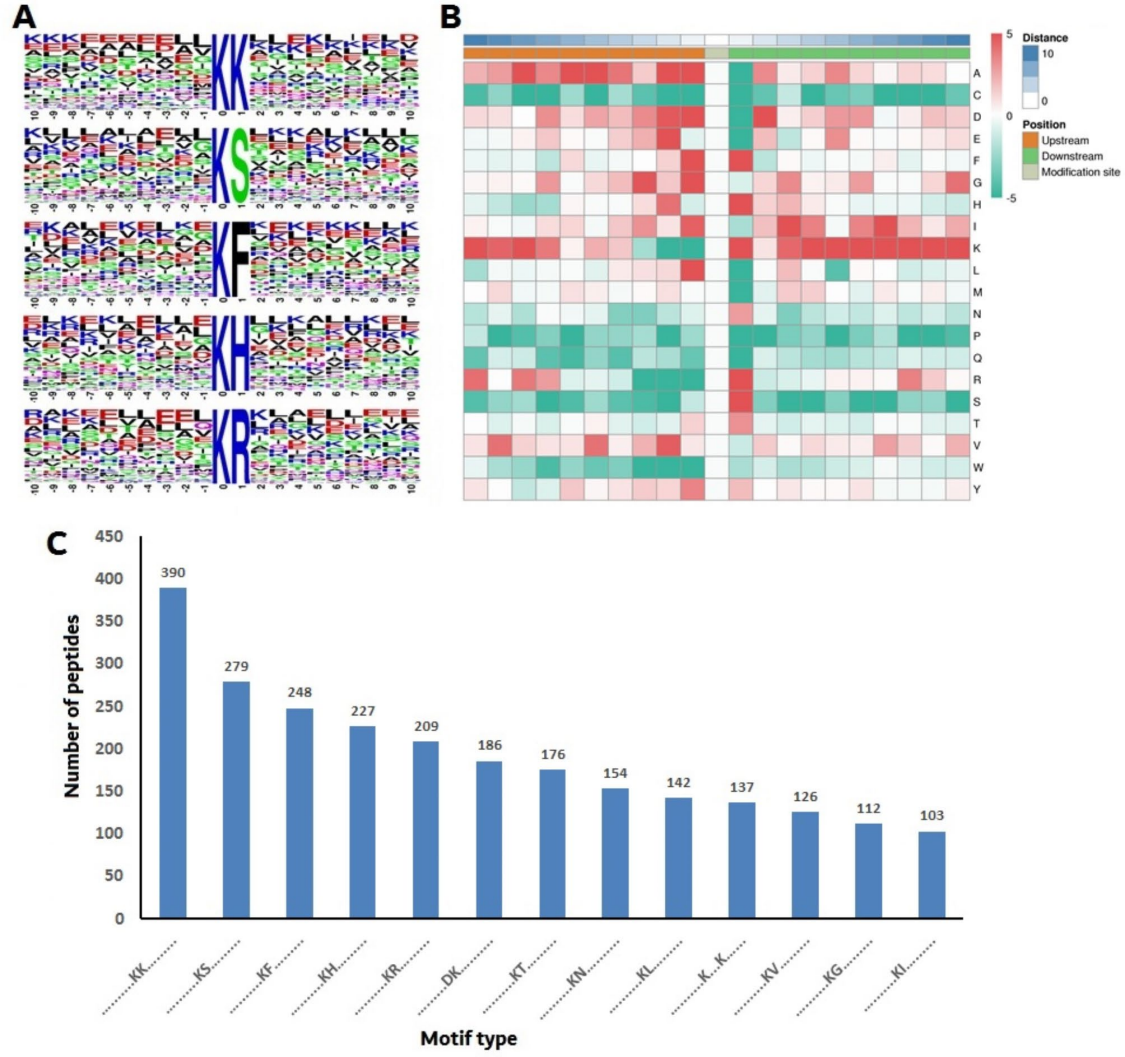
Characterization of acetylated peptides; **A** Probability sequence motifs of acetylation sites consisting of 20 residues surrounding the targeted lysine residue using Motif-X. thirteen significantly enriched acetylation site motifs were identified; **B** Heat map showing upstream (red) or downstream (green) of amino acid compositions around the acetylated lysine site (10 amino acids upstream and downstream of the acetylated lysine site); **C** Number of identified peptides possessing an acetylated lysine in each motif

Of all the acetylated peptides, 13 significantly enriched lysine acetylation site motifs from 2489 modified sites were identified within ten amino acids upstream and downstream positions of the acetylated lysine (Fig. 5). These motifs were KacK, KacS, KacF, KacH, KacR, KacT, KacN, KacL, KacV, KacG, KacI, KacD and Kac***K (Kac: acetylated lysine; *: residue of a random amino acid; Fig. 5A). Motif enrichment was also illustrated in the form of a heat map (Fig. 5B). The 2489 modified sites accounted for 86.6% of the sites identified according to the criteria of specific amino acid sequence. There was significant enrichment of lysine (K), serine (S), phenylalanine (F), histidine (H), arginine (R), threonine (T), asparagine (N), leucine(L), valine (V), glycine (G), and isoleucine (I) at position + 1 (75.4%, Fig. 5C). The first motif was remarkably conserved, and acetylated peptides with this motif accounted for approximately 15.7% of all identified acetylated peptides.

### Enrichment analysis of differentially acetylated proteins

**Fig. 6:**
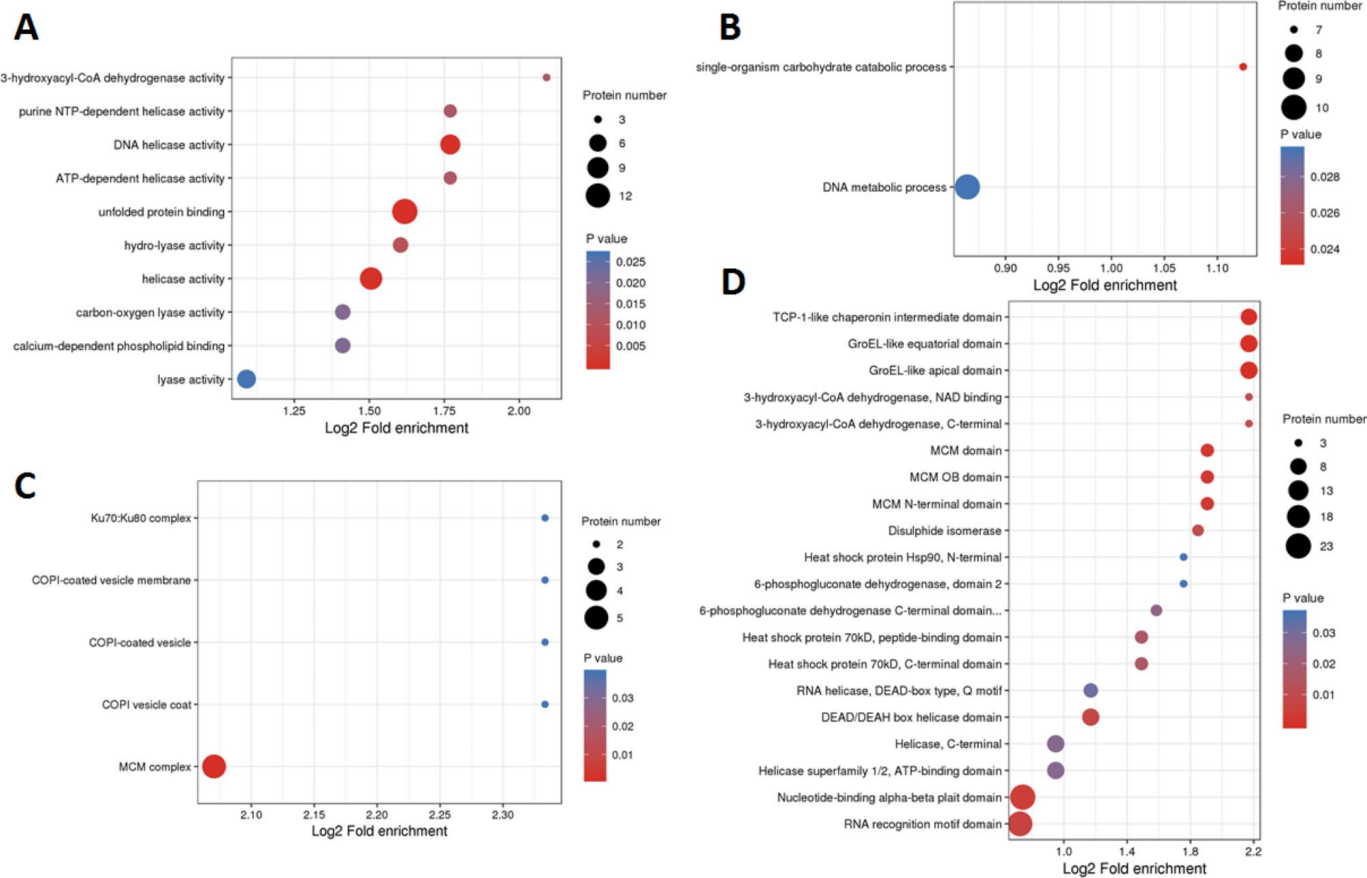
GO and domain enrichments of DAcPs; **A** Molecular function; **B** Biological process; **C** Cellular component **D** Protein domain. The x-axis was the rich factor which means the proportion of DAcPs in total proteins. The y-axis was the protein functional classification. Different colors of plots indicated different P values. Plot diameter represented DAcPs numbers in a GO term

In order to further explore the functions of the acetylated proteins during PPRV infection, GO enrichment (cellular component, biological process and molecular function), KEGG and protein domain analysis were performed for all identified proteins with differentially acetylated sites (Fig. 6 & Additional file 6: Table S4).

In the molecular function category, unfolded protein binding and helicase activity were found to be significantly enriched (Fig. 6A). Only two enriched biological processes (single-organism carbohydrate catabolic and DNA metabolic processes) were identified (Fig. 6B). The most enriched cellular component was minichromosome maintenance (MCM) complex (Fig. 6C). The KEGG database was used to identify pathways associated with the DAcPs. 17 DAcPs were involved in protein processing in endoplasmic reticulum (ER), and 12 of which were down-regulated. Protein domain analysis showed that a large proportion of DAcPs were associated with the nucleotide − binding domain, RNA recognition motif domain, GroEL-like domain, TCP-1-like chaperonin intermediate domain, MCM domain and DEAD/DEAH box helicase domain (Fig. 6D).

### Protein–protein interaction network analysis of differentially acetylated proteins

**Fig. 7:**
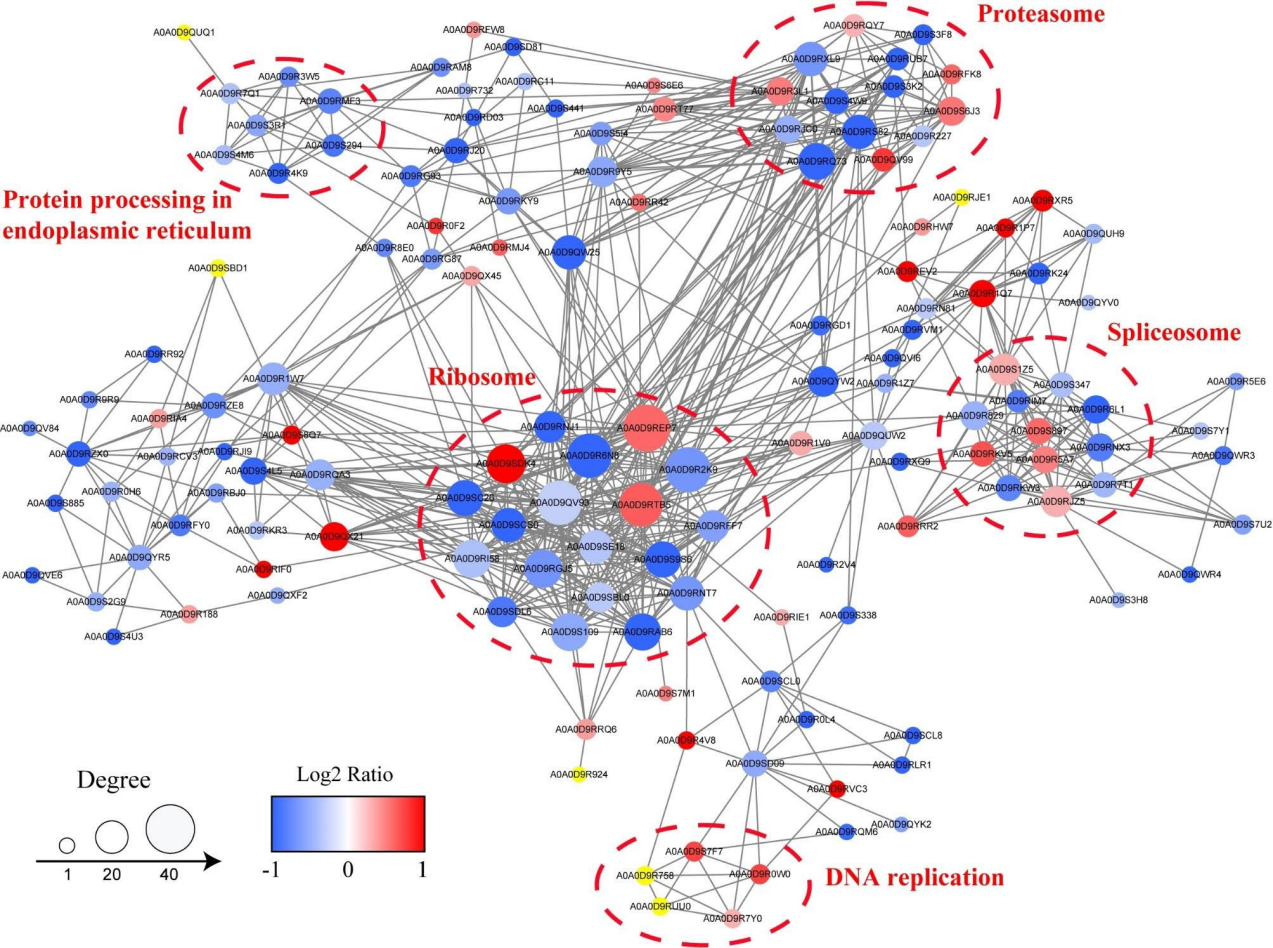
The protein-protein interaction network of DAcPs. Each node represented a protein, and each line indicates an interaction. Node colors represent fold change, and circle size represents the numbers of DAcPs. Red indicates upregulated DAcPs and blue indicates downregulated DAcPs

To better understand how acetylation regulates diverse metabolic processes and cellular functions, we assembled PPI networks of the identified modified proteins. In total, 147 DAcPs were mapped to the protein network database. A global network graph of these interactions was shown in Fig. 7 and Additional file 7: Table S5. As shown in Fig. 7, five highly connected subnetworks, namely, ribosomes, proteasomes, spliceosomes, protein processing in the ER, and DNA replication of DAcPs, were enriched. In the first subnetwork, 19 proteins with 96 Kac sites and 484 direct physical interactions participated in the ribosomal interaction, suggesting that they play key roles in protein synthesis. The second subnetwork is related to the 26 S proteasome and chaperone, and comprised 14 proteins with 75 Kac sites and 184 direct physical interactions. Among the 14 proteins, six subunits of the 26 S proteasome and five paralogous subunits of chaperonin containing the T-complex polypeptide-1 (CCT, also called TRiC) were identified as DAcPs in PPRV infection: proteasome 20 S subunit (PSMA4, PSMA5, and PSMB3), proteasome 19 S subunits (PSMC5, PSMD11 and PSMD13) and CCT subunit (CCT3, CCT4, CCT5, CCT6A and CCT7). Moreover, in the ribosome and proteasome subnetworks, a tight PPI network including 8 up-regulated proteins and 25 down-regulated proteins, was significantly enriched. Most proteins in the PPI network contained more than two acetylated sites. Overall, these results suggest complex interactions among acetylated proteins that may control the disease response or resistance during PPRV infection.

## Discussion

Pathogen infection can regulate biological processes of the host at various levels that can lead to major changes in the proteome, involved at transcriptional, post-transcriptional, translational, and post-translational levels. As a widespread PTM, lysine acetylation plays central roles in regulating multiple biological processes, particularly metabolism [5]. Recently, increasing instances of protein acetylation on lysine residues have been reported. Accumulating evidence suggests that lysine acetylation is a key molecular toggle for host antiviral responses and virus replication either directly or indirectly [25, 26, 32]. Despite these findings, protein acetylation remains an understudied aspect of viral infections due to the lack of knowledge regarding its temporal regulation during infection.

Although comparative transcriptome and proteomic profiling has been performed in bone marrow-derived dendritic cells (BMDCs) and peripheral blood mononuclear cells (PBMCs) stimulated with PPRV [33–35], little is known about lysine acetylation in cells infected with PPRV. In this study, we used acetylome analysis approaches based on affinity purification and LC–MS/MS to elucidate the effects of PPRV infection on the protein lysine acetylation profiles of Vero cells to gain insight into PPRV pathogenesis. This is the first systematic analysis of acetylation in Vero cells infected with PPRV, providing an important starting point for functional studies of acetylated proteins in response to PPRV infection. Among the DAcPs identified, vimentin and NCL were modified at multiple lysine acetylation sites. Vimentin, one of the most widely expressed intermediate filament proteins, can facilitate viral internalization, entry and replication by interacting with the proteins of porcine reproductive and respiratory syndrome virus (PRRSV), Japanese encephalitis virus, parvovirus, enterovirus 71, SARS-CoV, Bluetongue virus, dengue virus and transmissible gastroenteritis virus [36–44]. Rearrangement of cytosolic vimentin and the formation of vimentin cages around the viral factories in African swine fever virus and vaccinia virus infection were beneficial for preventing the movement of viral components into the cytoplasm and contributing to efficient assembly and replication of the virus [45, 46]. NCL also plays important roles in the entry, replication and intracellular transport of several viruses. Changes in NCL localization in virus-infected cells have been reported. NCL interacts with PPRV N protein and indirectly inhibits PPRV growth by stimulating the interferon (IFN) pathway [47]. In contrast, NCL facilitates the entry of influenza A viruses and enterovirus (EV)71 [48, 49]. Acetylated NCL is present in speckle structures in the nucleoplasm and co-localizes with the splicing factor SC35, suggesting that NCL is involved in pre-mRNA synthesis or metabolism [50]. Interestingly, differentially expressed DAcPs were rare in PPRV-infected cells. These results suggest that the roles of acetylated proteins in PPRV entry and replication are determined by their acetylation levels.

By assessing enriched motifs in the acetylated peptides, we found that the acetylation sites identified in this study had similar sequence motifs to those in the previous reports [26, 51, 52]. We also observed that the residues flanking acetylation sites were highly enriched in lysine at the + 1 position, and frequently contained K, H and R amino acids. These results indicated that the lysine residue of a polypeptide with a K, H or R amino acid at the + 1 position is the preferred substrate for lysine acetyltransferase.

GO functional classification analysis showed that many of the identified acetylated proteins in PPRV-infected cells were related to the MCM complex. The MCM complex is required for successful viral genome replication [53]. The complex composed of MCM2 to MCM7 (MCM2–7) is a part of the viral chromatin at the replication origin in the terminal repeat region, unwinds DNA to initiate replication and acts as a helicase on elongating DNA [54, 55]. However, acetylated MCM3 inhibits the initiation of DNA replication and cell cycle progression [56]. The ER is critical for protein synthesis and maturation and relies on many molecular chaperones that assist in protein folding and assembly. Virus infection can alter ER and activate the unfolded-protein response to facilitate viral replication [57, 58]. The CCT chaperonin mediates protein folding and is essential for the assembly of functional viral replicons. CCT, a molecular chaperonin [59, 60] involved in synergistic immunity [61], apoptosis [62, 63], and cell-cycle regulation [64], mediates protein folding and is essential for the assembly of functional viral replicons. Recently, an increasing number of studies have reported that molecular chaperones play an important role in the life cycle of viruses, including viral entry, replication, transcription, translation, virion assembly, and even viral cell-cell movement [65–69]. In the present study, two different sites were acetylated in six paralogous subunits of CCT, and the acetylation levels of these CCT subunits were decreased in PPRV-infected cells, suggesting that an alteration of transcriptional response triggered by chaperones has happened, which may be able to alter PPRV infection. Based on the results of this study, acetylation of chaperonins and cytoskeletal proteins may play a vital role in viral infection; however, the relationship between PPRV infection and DAcPs requires further investigation. A comprehensive study on the relationship between PPRV and these chaperones will open new directions to understand of pathogenesis, prevention and control of PPRV infection.

PPIs are critical for various biological processes. This study provides the first global PPI network of acetylated proteins induced by PPRV infection. Various cellular interactions are modulated at the acetylation level upon viral infection. Indeed, we identified subnetworks of ribosomes and proteasomes enriched in this study, indicating a critical role of these acetylated proteins in response to PPRV infection. The ubiquitin-proteasome system (UPS) regulates the expression levels of cellular proteins by ubiquitination of protein substrates followed by their degradation via the proteasome. Viruses subvert or manipulate this cellular machinery to favor viral propagation and to evade the host immune response. The UPS participates in viral propagation and acts as a double-edged sword in viral pathogenesis [70–72]. 26 S proteasome is a major molecular complex responsible for protein degradation in eukaryotes. The acetylation levels of four UPS-linked proteins (PSMA4, PSMA5, PSMC5 and PSMD13) identified as DAcPs following PPRV infection were up-regulated, suggesting that viral proteins may be degraded by UPS to limit PPRV infection. The proteasome subnetwork is consistent with KEGG analysis. Mutual corroboration between the results of subnetwork proteasome and KEGG pathway enrichment confirmed the reliability of analysis.

Histone modification is a typical epigenetic modification. Increasing evidence has indicated that histone acetylation plays an important role in virus infection. Several previous studies have reported virus-induced changes in histone lysine acetylation sites, such as adenovirus [73], Borna disease virus [24, 74], Influenza virus [23, 75], HIV [76], Zaire Ebolavirus [77], bovine herpesvirus [78], and parvovirus [79]. In this study, nine histone lysine acetylation sites were significantly differentially regulated, eight of which were down-regulated. This result hints that histone acetylation modification may also exert certain functions in Vero cells upon PPRV infection. Intensive investigations are required to explore the precise biological function of these acetylated proteins in PPRV infection.

## Conclusions

In summary, our findings provide quantitative profiling of the acetylome of PPRV-infected Vero cells. We identified 304 proteins with 410 acetylation sites, that were significantly differentially acetylated in response to PPRV infection. These DAcPs primarily participated in carbohydrate catabolism and DNA metabolism, suggesting that intracellular activities are extensively altered after PPRV infection. The PPI network further indicated that various chaperones and ribosomal processes were modulated by acetylation. To the best of our knowledge, this is the first study of acetylome of Vero cells infected with PPRV. This work provides an important starting point for future studies on the acetylated proteins involved in the host response to PPRV.

### Electronic supplementary material

Below is the link to the electronic supplementary material.

**Additional file 1: Figure S1**. Western blotting with an anti-pan acetyllysine antibody in response to PPRV infection in Vero cell

**Additional file 2: Figure S2**. **A** Mass error of all identified peptides; **B** Pearson’s correlation of protein quantitatio

**Additional file 3: Tables S1**. Quantitative acetylated sites on proteins

**Additional file 4: Tables S2**. Summary of differentially quantified acetylated sites and proteins in different groups

**Additional file 5: Tables S3**. Classification of differentially acetylated proteins

**Additional file 6: Tables S4**. Enrichment of differentially acetylated proteins

**Additional file 7: Tables S5**. The identified acetylated proteins used for PPI

## Data Availability

Not applicable.

## Abbreviations

PPR: *Peste des petits ruminants*
PPRV: *Peste des petits ruminants* virus
DAcPs: differentially acetylated proteins
PPI: Protein-protein interaction
PTM: Protein post-translational modification
GO: Gene Ontology
KEGG: the Kyoto Encyclopedia of Genes and Genomes
Kac: acetylated lysine
CCT: chaperonin containing T-complex polypeptide-1
