# Supplementary figures and images for: Quantitative analysis of acetylation in peste des petits ruminants virus-infected Vero cells
Source: Virol J. 2023 Oct 10;20:227. doi: 10.1186/s12985-023-02200-1 (PMC10563215; doi:10.1186/s12985-023-02200-1)

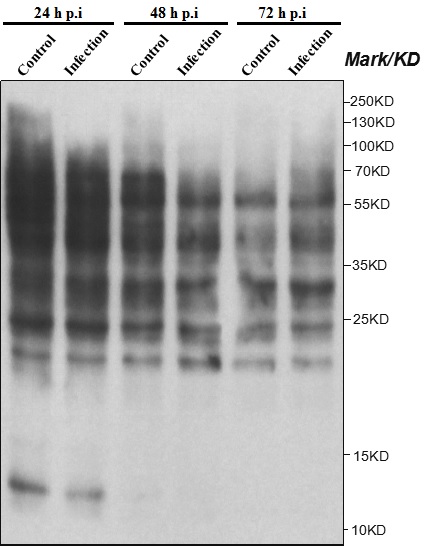

Supplement: Supplementary file 1 — Additional file 1: Figure S1. Western blotting with an anti-pan acetyllysine antibody in response to PPRV infection in Vero cell [file 12985_2023_2200_MOESM1_ESM.jpg]

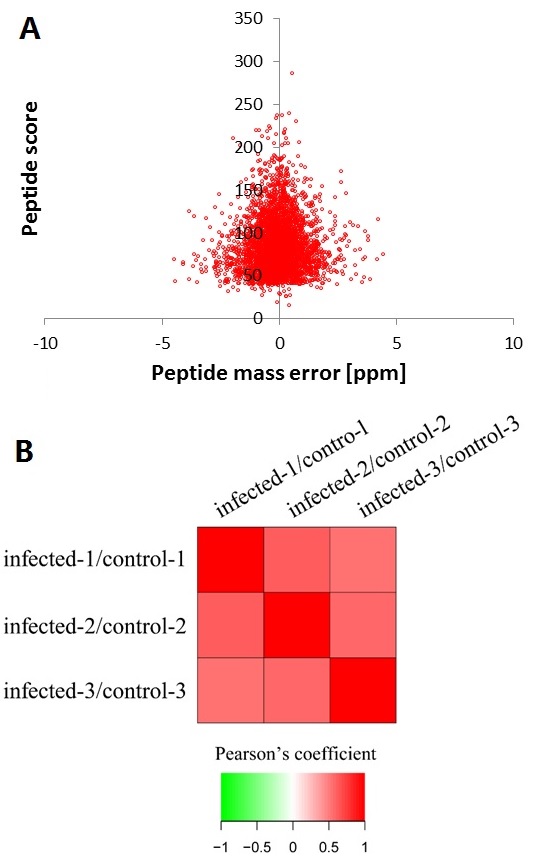

Supplement: Supplementary file 2 — Additional file 2: Figure S2. A Mass error of all identified peptides; B Pearson’s correlation of protein quantitatio [file 12985_2023_2200_MOESM2_ESM.jpg]
